# The Effect of Massage Therapy on Autonomic Activity in Critically Ill Children

**DOI:** 10.1155/2014/656750

**Published:** 2014-12-21

**Authors:** Ling Guan, Jean-Paul Collet, Nataliya Yuskiv, Peter Skippen, Rollin Brant, Niranjan Kissoon

**Affiliations:** ^1^Department of Pediatrics, BC Children's Hospital, University of British Columbia, Vancouver, BC, Canada V6T 1Z3; ^2^Department of Medicine, Faculty of Medicine, University of British Columbia, Vancouver, BC, Canada V6T 1Z3; ^3^Child and Family Research Institute, 950 West 28th Avenue, Vancouver, BC, Canada V5Z 4H4; ^4^Division of Critical Care, BC Children's Hospital, University of British Columbia, Vancouver, BC, Canada V6H 3V4; ^5^Department of Statistics, University of British Columbia, Vancouver, BC, Canada V6T 1Z4

## Abstract

*Objectives*. Our main objective was to describe the effect of foot and hand (F&H) massage on the autonomic nervous system (ANS) activity in children hospitalized in a pediatric intensive care unit (PICU); the secondary objectives were to assess the relationship between ANS function and the clinical severity and to explore the effects of repeated massage sessions on the ANS. *Methods*. Design was a descriptive experimental study. Intervention was single or six session(s) of F&H massage. ANS function was assessed through the frequency-domain analysis of heart rate variability. Main metrics included high and low frequency power (HF and LF), HF + LF, and LF/HF ratio. *Results*. Eighteen children participated in the study. A strong Spearman's correlation (*ρ* = −0.77) was observed between HF + LF and clinical severity. During massage, the parasympathetic activity (measured by HF) increased significantly from baseline (*P* = 0.04) with a mean percentage increase of 75% (95% CI: 20%∼130%). LF increased by 56% (95% CI: 20%∼92%) (*P* = 0.026). Repeated sessions were associated with a persistent effect on HF and LF which peaked at the second session and remained stable thereafter. *Conclusions*. HF + LF is positively correlated with clinical severity. F&H massage can improve the ANS activity and the effect persists when repeated sessions are offered.

## 1. Introduction

Children in pediatric intensive care units (PICU) face multiple stressors and are at high risk of developing systemic inflammatory response syndrome (SIRS) and life-threatening multiple organ dysfunction syndrome (MODS) [1]. The autonomic nervous system (ANS) whose main function is preserving body homeostasis through tight control of stress reaction and inflammatory response [2] is seriously impaired in ICU stressful situations [3, 4], with the typical association of an overstimulated sympathetic nervous system (SNS) and a blunted parasympathetic nervous system (PNS) [2, 5, 6]. Autonomic activity can be assessed in a noninvasive and dynamic way at heart level by measuring the variability of the heart rate (HRV) [7–9]. Landmark studies in adults and children have documented the strong relationship between depressed ANS (i.e., low HRV) and further clinical deterioration and death [10–13]. Conversely, the recovery phase from SIRS or sepsis is characterized by the improvement of HRV or “recoupling of biological oscillators” and a more favourable prognosis [11, 12, 14].

From a therapeutic perspective the anti-inflammatory property of the cholinergic pathway attracted a lot of interest [2, 15–17]. In mice with experimental sepsis the direct stimulation of the vagus nerve is associated with a dramatic reduction of inflammatory cytokines (TNF-alpha) and enhances survival [15, 17]. Overall, restoring the ANS function and in particular the PNS activity appears to be a critical step in the recovery phase of seriously ill patients.

Different kinds of interventions such as exercise, diaphragmatic breathing, relaxation, or massage have been shown to influence the ANS [18–21]. Several studies have already demonstrated the effects of massage interventions to regulate the overactive SNS and blunted PNS in different situations [22, 23]. In the critical care environment with limited body access, massage of the limb extremities has been shown to reduce patients' stress and pain [24, 25]. The main objective of this study was to assess whether foot and hand (F&H) massage could improve the ANS function of critically ill children and to identify the factors that may affect the response. The secondary objectives consisted of assessing the relationship between baseline HRV and clinical severity and exploring the effects of repeated massage sessions on the ANS. This study which aimed to document the effects of massage on the ANS is considered the fundamental preliminary step before going further to assess possible biological and clinical impacts in critically ill children.

## 2. Materials and Methods

### 2.1. Design

This study is a short-term descriptive prospective experimental study. All participants received one session of F&H massage and a subgroup received six sessions. The decision to administer six sessions was made according to the critical care plan and duration of stay in the unit.

The study was approved by the Ethics Committee of the University of British Columbia and Children's & Women's Health Center Clinical Research Ethics Board.

### 2.2. Subjects

All children admitted to the PICU were eligible, but we only included subjects whose condition was stable, defined as no change in use of inotropic medications for at least six hours. Patients with arrhythmia were excluded along with those treated by Dexmedetomidine hydrochloride (Precedex), which seriously affects the HRV [29, 30]. Written informed consent was obtained from all subjects' parents/legal guardians and written assent was from subjects between 7 and 18 years of age.

### 2.3. Assessment of Demographic and Clinical Information

Relevant demographic and clinical information (diagnosis, organs dysfunction, and medications) was collected from the patients' medical charts. The severity of illness and organ dysfunction was assessed with the Pediatric Logistic Organ Dysfunction (PELOD) scoring system. The PELOD score is calculated through the assessment of six key organ functions: cardiovascular, respiratory, hematological, neurological, renal, and hepatic. The total score is 71 and a higher score reflects a more severe condition. The PELOD scoring system showed high reliability with a kappa coefficient ranging from 0.73 to 1 [31]. It also has a good calibration (goodness of fit, Hosmer-Lemeshow value of 4.03, *P* = 0.54) [31] and excellent discrimination properties (area under the receiver operating characteristic curve = 0.91 and 0.86) [31, 32]. The PELOD score is commonly used in clinical practice and research to describe the clinical severity of critically ill children [31–33].

### 2.4. Massage Intervention

The massage intervention was administered at the earliest convenient time after consent between 9:00 am and 7:00 pm. Each session was of 20~30 minutes long and the time interval between two sessions in the subgroup of children who received six sessions was 1 to 1.5 hours. This fine variation of time between massage sessions was related to the necessary medical procedures. The massage process consisted of six steps: introduction touch, right hand, left hand, right foot, left foot, and closure. Eleven registered massage therapists (RMTs) were involved and received special training to standardize practice. The RMTs recorded the precise beginning and ending time of the procedure and reported the child's reaction as well as the environment in the ward. A video camera was used to check the child's reaction and the environment to provide a more informed interpretation of what could influence the patient's HRV; the video was also used for controlling the good application of the massage protocol by RMTs. The videotaping was approved by ethics. All people working in the PICU unit as well as the patients/parents were informed of the videotaping but did not have to sign a special consent form. These videos were only used for research and only researchers directly involved in the project could access them.

### 2.5. Assessment of ANS Activity

The ANS activity was assessed using the frequency-domain analysis of heart rate variability technique [8]. HRV data were extracted from the 24-hour electrocardiogram (ECG) recording and analyzed with the* Acqknowledge* software (BIOPAC Systems, Inc., Goleta, CA, USA). In the PICU, the ECG recording at each bed is transferred to the central station computers where it is stored for a couple of days. With the information (starting and end time of the massage intervention) from the therapists' notes, the appropriate sequence of ECG raw data was extracted from the central computer through a special program and transferred to the study computer. The sampling rate of ECG acquisition was 250 Hz. HRV data were analyzed for 5 minutes before massage as baseline, every 5 minutes during massage (4–6 times), and 5 minutes after massage. Artifacts and ectopic beats were removed and replaced manually by an extrapolation of the beat to beat interval calculated from the 3 beats preceding and the 3 beats following the artifact to ensure that only normal R-R intervals were kept in the HRV analyses. The HRV data were analyzed according to the Task Force of the European Society of Cardiology and the North American Society of Pacing and Electrophysiology Guidelines [8]. The researchers edited and double-checked the recordings and reanalyzed the data three times to ensure consistency.

At each assessment, the frequency-domain indexes of HRV were calculated from the power spectrum after fast Fourier transformation and expressed in milliseconds squared per hertz. High frequency (HF) power (0.15–0.4 Hz) represents PNS activity; low frequency (LF) power (0.04–0.15 Hz) reflects a mix of SNS and PNS modulations; and HF + LF reflects the variability of heart rate mostly related to the autonomic modulation. The very low frequency (VLF) power (<0.04 Hz), a nonharmonic component without coherent properties [8], was not considered in the analysis. Additionally, HF and LF were also measured as normalized units: HF/(LF + HF) for normalized HF (HF n.u.) and LF/(LF + HF) for normalized LF (LF n.u.). The normalized values are relative values of each power component that minimize the effect of changes in total power when comparing changes of HF and LF (absolute values) at different times; normalized values emphasize the controlled and balanced behavior of the two branches of ANS [8, 34, 35]. The change in LF to HF ratio was also assessed as a marker of sympathetic-parasympathetic balance [8].

### 2.6. Statistical Analysis

Baseline HF values of study subjects (as a marker of PNS activity) were compared with the age-specific normal values from the literature (Table 1). To assess the relationship between baseline HF + LF and clinical severity (PELOD score), the Spearman rank correlation coefficient (*ρ*) was calculated.

All study subjects contributed to assessing the effect of one massage session. HRV assessment during the 5 minutes before massage was set as “baseline”; HRV for each five-minute interval during massage was analyzed and the mean of those intervals was set as “during-massage” effect; finally, the five-minute interval after massage was used to assess the remaining effect of massage. In order to control for the huge variation in HRV data among subjects (from 0.01 msec^2^/Hz to 500 msec^2^/Hz), the original data of HF and LF were natural logarithmically transformed (ln) for statistical analyses [36–38]. Repeated measures analysis of variance (RM-ANOVA) was used to assess the changes of HRV variables over the course of the massage session, with assessment of pairwise comparisons. Mean percentage change with 95% confidence interval (95% CI) was computed to describe the change of HF and LF from baseline to during- and after-massage periods. Graphics represent the effect of repeated massage sessions.

All statistical analyses were performed with IBM SPSS statistics 20 software (SPSS Inc., Chicago, IL).

## 3. Results

**Figure 1:**
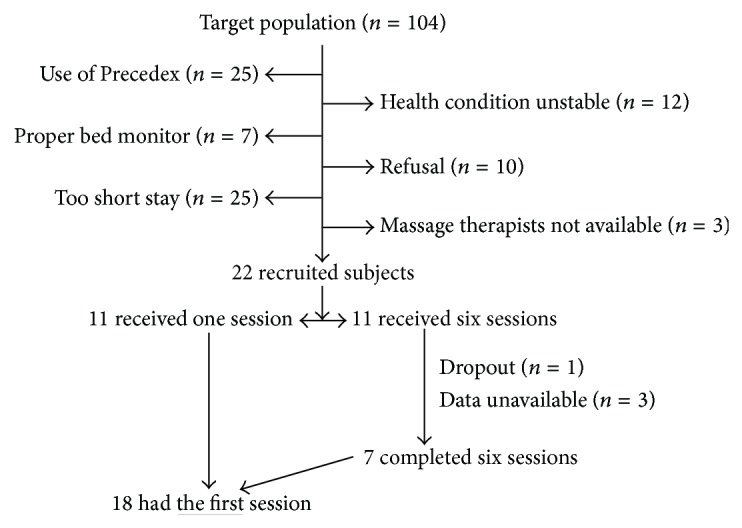
Study recruitment flow chart.

Over the period from April 2011 to February 2012, we contacted 104 eligible subjects admitted to the PICU at BC Children's Hospital, Vancouver, Canada; however due to incompatible drugs (Precedex, *n* = 25), short duration of stay (*n* = 25), unstable health condition (*n* = 12), unavailable cardiac recording (*n* = 7), refusal (*n* = 10), and the nonavailability of RMTs (*n* = 3), only 22 PICU subjects were included in the study (Figure 1). The 22 subjects received one session of massage and a subgroup of 11 subjects received an extra five sessions. At the time of massage administration, among the 22 subjects, 11 were treated by morphine, 8 were sedated with midazolam or fentanyl, 4 received catecholamines (3 with epinephrine and 1 with dopamine), and none was mechanically ventilated. However, HRV data of 4 subjects could not be used because of numerous extrasystole beats (*n* = 1) or technical reasons at the central station (*n* = 3), leaving 18 subjects' data for the analyses (11 with one session and 7 with six sessions) (Figure 1). The 18 subjects were diagnosed on the admission to PICU with, respectively, pneumonia/bronchiolitis (*n* = 6), sepsis/septic shock (with/without leukemia or acute renal failure) (*n* = 4), cardiac postoperative care (*n* = 2), burns (*n* = 1), trauma (*n* = 1), status epilepticus (*n* = 1), stridor (*n* = 1), congenital heart disease plus respiratory infection (*n* = 1), and pulmonary hypoplasia plus upper gastrointestinal bleeding (*n* = 1).

### 3.1. HRV and Clinical Severity

HF for PICU patients was at least two standard deviations below the age-specific normal range (Table 1), with extreme reduction in some cases: HF was only 1/1000 of the normal values.

**Figure 2:**
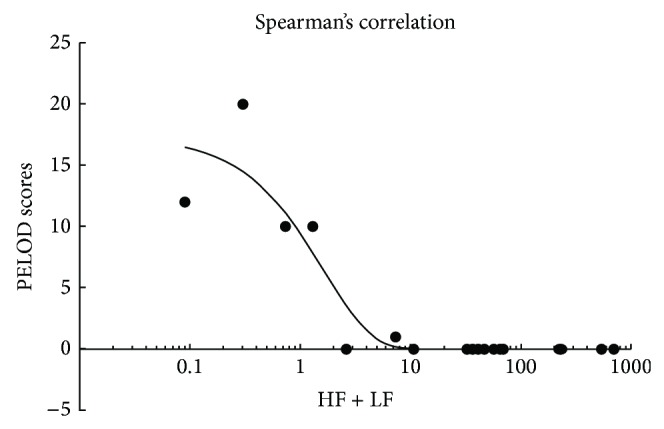
Correlation between HF + LF values and clinical severity score in patients in pediatric intensive care unit. PELOD scores: Pediatric Logistic Organ Dysfunction scores; HF + LF: high and low frequency power of heart rate variability. The Spearman rank correlation coefficient *ρ* = −0.77, 95% CI: −0.91, −0.47; *P* < 0.001. In the figure, HRV data (*x*-axis) have been logarithmically transformed (i.e., HRV data).

Table 2 shows at baseline the distributions of HF + LF values and the PELOD score of the 18 subjects with their diagnoses. The Spearman rank correlation coefficient (*ρ*) was −0.77 with 95% CI from −0.91 to −0.47 (*P* < 0.001) (Figure 2). Patients with the highest PELOD score had the lowest absolute HF + LF values; the only exception was a patient with status epilepticus who showed low HF + LF data with a PELOD score of 0 (Table 2).

### 3.2. Effect of One Massage Session on Autonomic Activity

The changes of lnHF from baseline to during- and after-massage periods were significant (repeated ANOVA: *F* = 3.94; *P* = 0.03) with pairwise significance (*P* = 0.04) between baseline and during-massage (Table 3). Similar findings were found with lnLF (repeated ANOVA: *F* = 3.39; *P* = 0.046) with significant change (*P* = 0.026) from baseline to during-massage (Table 3). In contrast, the mean of normalized HF and LF as well as LF/HF remained stable at three time points (Table 3). The distribution of LF/HF, however, was centralized, with a shift toward the normal range for pediatric standards (0.73 +/− 0.08~2.43 +/− 0.88) [26, 27, 39]: higher ratios decreased and lower ones increased, while values originally in the normal range remained stable (not shown).

**Figure 3:**
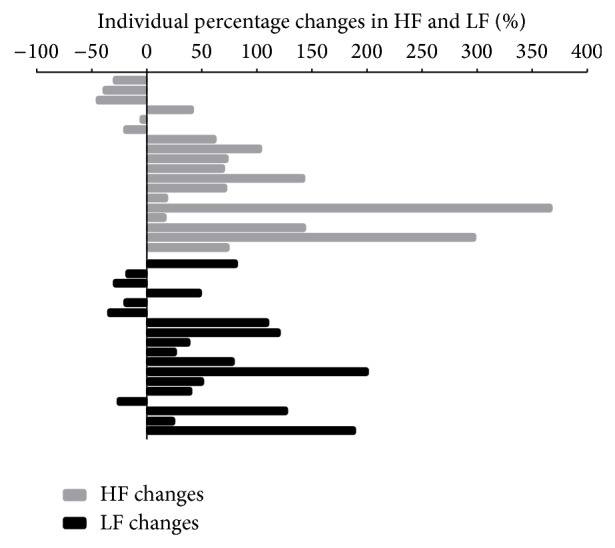
Individual percentage change of heart rate variability during massage. Mean percentage increase of HF during massage (significant): 75% (95% CI: 20% TO 130%). Mean percentage increase of LF during massage (significant): 56% (95% CI: 20% TO 92%).

Thirteen subjects exhibited an important increase of HF during massage, especially 2 of them, while 5 subjects showed a minor decrease. Overall, the mean percentage increase in HF during massage was 75% (95% CI: 20%~130%, median increase of 67%) (Figure 3). LF also increased significantly during massage compared to baseline: 56% (95% CI: 20%~92%; median increase of 45%) (Figure 3). Finally, HF + LF increased during the massage by a mean of 54% (95% CI: 24%~84%; median increase of 53%). During the five minutes after massage we observed the remaining effect with an average HF increase of 34% (95% CI: −11%~78%; median increase of 17%) compared to baseline; more specifically, 10 subjects showed a persistent increase of HF compared to baseline, with values >150% in three (not shown). Similar findings were found for LF after massage (mean increase of 37% with 95% CI: −0.5% to 75%; median increase of 32%).

Finally, the median increases of HF, LF, and HF + LF during massage in the 4 sickest subjects (PELOD scores > 10) were higher than the ones observed in milder cases (the 14 patients with PELOD scores of 0 and 1): 84% versus 57% for HF, 81% versus 40% for LF, and 81% versus 48% for HF + LF, respectively; these differences however did not reach statistical significance (Mann-Whitney *U* test *P* ≈ 0.38 for all three tests).

### 3.3. Cumulative Effect of Repeated Massage Sessions on Autonomic Activity

**Figure 4:**
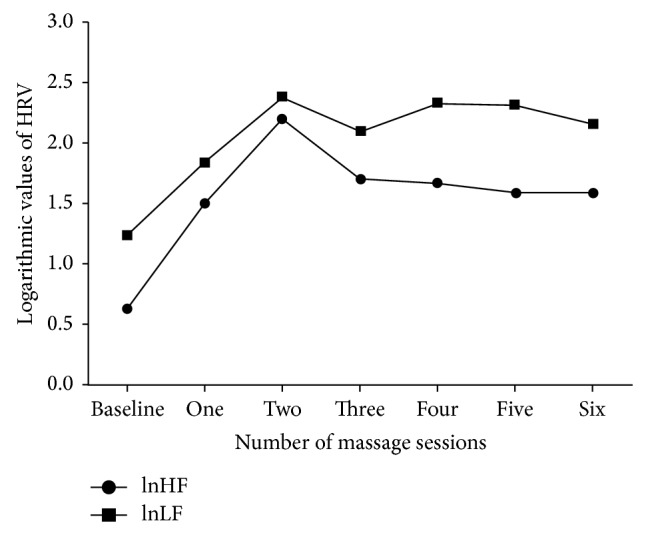
Heart rate variability changes during repeated massage sessions. lnHF: natural logarithm of the high frequency power; lnLF: natural logarithm of the low frequency power. Mean lnHF (with SE) at baseline and during six massage sessions: 0.63 (1.18), 1.51 (1.23), 2.21 (1.2), 1.7 (1.13), 1.67 (1.23), 1.59 (1.28), and 1.59 (1.22). Mean lnLF (with SE) at baseline and during six massage sessions: 1.24 (1.24), 1.84 (1.26), 2.39 (1.26), 2.1 (1.17), 2.33 (1.23), 2.32 (1.09), and 2.16 (1.19).

Figure 4 describes the changes of lnHF and lnLF over the course of six massage sessions. From a mean baseline value of 0.63, lnHF increased during the first session and reached 2.21 during the second session; it then decreased at the third session (1.7) to remain steady until the sixth session (1.59); after all six sessions of massage, the lnHF value (1.01) was still much higher than the baseline value. A similar pattern was also shown for lnLF (from baseline 1.24 to 2.39 at the second session and then stable until the end). The LF/HF ratio remained within normal ranges during the full course of six massage sessions (between 2.26 and 3.73, not shown).

## 4. Discussion

In this study we found low baseline HRV values as a sign of impaired autonomic control among all patients studied in the PICU and greater impairment in patients with sepsis, MODS, and leukemia. We also found a strong negative correlation (*ρ* = −0.77) between PELOD scores as a marker of clinical severity and HF values (Table 2 and Figure 2). Similar findings have been reported in a prospective study of 30 children with sepsis [10] which showed that LF and HF were considerably lower in patients with septic shock as compared to less severe sepsis. Our results therefore imply that HRV may be used as a marker of clinical severity in PICU patients. The only exception in our study is one child hospitalized for status epilepticus whose HRV was very low (2 msec^2^/hz) in contrast to a PELOD score of 0. This particular profile may reflect alteration of central nervous system activity by complex seizures [40] with subsequent loss of ANS modulation. These results need to be further studied to determine the possible use of HRV monitoring as a real-time marker of clinical severity and possibly an objective indicator of brain compromise.

Our study also shows that in a highly stressful environment characterized by sympathetic dominance, massage therapy is able to stimulate the PNS and the overall ANS activity (Table 3, Figure 3). This result is in agreement with previous studies in preterm babies [41, 42]. The increase of HF + LF power during massage is certainly a positive sign showing an improvement in autonomic system modulation of the stress response. Indeed, during the massage intervention the ratio of LF to HF improved and tended towards the normal range. Besides a simple regression to the mean, this observation may also be interpreted as a possible physiological effect of massage in favor of homeostasis restoration.

The four patients with highest PELOD (>10) scores and lowest HRV values (Table 2) showed a higher increase in HRV than the 14 less severe cases (PELOD score of 0 or 1) during massage therapy. This indicates a possible more beneficial effect of massage among subjects with highest severity scores and stress state. Other studies have already reported the positive effect of massage in patients with leukemia to improve paraneoplastic autonomic neuropathy [43] and some side effects of chemotherapy [44]. In addition, in preterm babies massage reduced the occurrence of late-onset sepsis and decreased the duration of hospital stay [45].

Unexpectedly, four subjects with PELOD score of 0 (hospitalized for trauma, burns, respiratory distress/pneumonia, and RSV bronchiolitis) showed a slight decrease in HF and LF during the massage intervention. This decrease in HRV power was considered a possible negative reaction to massage therapy; the video recording showed that these children became irritated or bored during the course of massage. However it is possible that these patients felt greater stress as a result of the massage pressure. This contention is supported by Diego and Field who reported that the effect of massage may be related to the intensity of the pressure: subjects who received light pressure massage exhibited a SNS response (arousal), while moderate pressure stimulated the PNS [20]. This question deserves further studies with precise videotaping of the children's reactions during massage and concomitant assessment of the massage pressure.

The analysis of repeated massage sessions practiced at 60- to 90-minute time intervals showed that the maximum effect peaked at the second session (lnHF = 2.2 and lnLF = 2.39) and remained stable thereafter (Figure 4). If confirmed, this may support the implementation of repeated massage interventions over 24 hours in the PICU to stimulate HF + LF power and help coping with acute stress, similar to data in mice with sepsis where vagus nerve stimulation reduced the secretion of TNF-alpha and improved the outcomes [15, 17]. These results are also consistent with a study in preterm infants where repeated massage (three periods of 15 minutes per day for 5 days) increased PNS function [42].

These results should be interpreted with caution, however, because of the small sample size and the before-after design without controls. The time sequence of the changes in HRV parameters in relationship to the massage, however, strongly suggests that these changes are related to the intervention. The six massage interventions were administered over 8 to 10 hours during daytime, and thus our findings cannot be extrapolated to different numbers, frequencies, and other times of the day and night. Another limitation is the absence of direct data regarding the sympathetic function due to PICU restriction for measuring the preejection period (PEP), a specific marker of SNS activity [46]. Furthermore, most children were administered catecholamines and morphine which may modulate ANS activity, therefore affecting the individual's HRV values and possibly reducing the magnitude of the massage effect. The changes in HRV during the course of massage, however, were observed under steady drug exposure and the bias, if any, would work against the hypothesis, as drugs will likely decrease the ANS response to massage.

As a demonstration project to document the effect of massage on ANS in PICU children, our study did not aim to assess the impact of massage on clinical or biological parameters and did not use a sham control group. In this extreme situation the fact that F&H massage could improve the autonomic function is noteworthy. The study results are presently used for planning future formal sham controlled trials.

## 5. Conclusions

Overall, our study generates new information regarding the effectiveness of massage intervention in critically ill children and the feasibility of conducting repeated massage sessions in the PICU. Besides confirming the strong correlation between autonomic functions and disease severity our study shows that single session of F&H massage can modulate overall autonomic function. Another important result is the demonstration that the ANS remains receptive to repeated sessions when they are administered at a pace of about 1 to 1.5 hours during daytime. More studies are warranted to assess the effects of massage on biological and clinical outcomes in critically ill children.

## Figures and Tables

**Table 1:** Heart rate variability for critically ill children compared with normal values from the literature [26–28].

Age of the 18 PICU subjects (number of subjects)	HF of PICU subjects, ms^2^/Hz	HF normal range, ms^2^/Hz [26–28]	
		Mean	SD
2 months (*n* = 1)	2.6	25.03	2.36
5 months (*n* = 1)	22.4	25.03	2.36
8 months (*n* = 2)	28.3	36.6	3.03
10 months (*n* = 1)	25.9	36.6	3.03
2.5 years (*n* = 7)	106.5	407.48	3.56
5 years (*n* = 1)	0.04	398	377
6 years (*n* = 1)	0.13	398	377
8 years (*n* = 1)	8.35	469	275
12 years (*n* = 1)	0.07	285.5	272
14 years (*n* = 1)	9.17	285.5	272
17 years (*n* = 1)	0.36	285.5	272

PICU: pediatric intensive care unit; HF: high frequency.

**Table 2:** Heart rate variability and pediatric logistic organ dysfunction scores.

HF + LF, ms^2^/Hz		Age	Gender	Diagnosis (subject number)	PELOD scores
>100	700.98	2.5 y	F	Pneumonia, respiratory distress	0
	534.14	2.5 y	M	Postoperation (frontal lobe tumor)	0
	233.88	2.5 y	M	Pneumonia, respiratory distress	0
	222	8 m	M	RSV bronchiolitis	0

10–100	68.44	5 m	M	Stridor	0
	64.78	2.5 y	M	Pneumonia	0
	56.2	8 y	M	Pectus excavatum repair	0
	46.21	10 m	M	Respiratory infection, congenital heart disease	0
	42.01	8 m	M	Pulmonary hypoplasia plus upper gastrointestinal bleeding	0
	32.23	2.5 y	F	Pneumonia	0
	36.71	14 y	M	Burns	0
	10.58	2 m	M	RSV bronchiolitis	0

1–10	7.26	2.5 y	M	Trauma	1
	2.63	2.5 y	F	Status epilepticus	0
	1.3	17 y	F	Sepsis shock, presumed pyelonephritis, and vesicoureteral reflux	10

<0.1	0.73	6 y	F	Acute renal failure, sepsis shock, and respiratory distress	10
	0.3	5 y	F	Acute lymphoblastic leukemia, septic shock	20
	0.09	12 y	F	Acute myeloid leukemia, *E. coli* sepsis	12

PELOD scores: Pediatric Logistic Organ Dysfunction scores; HF + LF: high and low frequency power of heart rate variability.

**Table 3:** One-session massage effect on heart rate variability.

	HRV values Mean (95% CI) (*n* = 18)			*P* values			
	Baseline (B)	During massage (D)	After massage (A)	Repeated measures ANOVA	Pairwise comparison		
					B~D	B~A	D~A
lnHF	1.63 (0.26 to 3.01)	2.02 (0.65 to 3.39)	1.69 (0.38 to 2.99)	0.03	0.04	1	0.05
lnLF	2.44 (1.13 to 3.76)	2.78 (1.5 to 4.07)	2.59 (1.39 to 3.78)	0.046	0.026	1	0.34
Normalized HF, n.u.	0.33 (0.23 to 0.44)	0.33 (0.24 to 0.42)	0.32 (0.22 to 0.43)	0.9	NA	NA	NA
Normalized LF, n.u.	0.67 (0.56 to 0.77)	0.67 (0.58 to 0.76)	0.68 (0.57 to 0.78)	0.9	NA	NA	NA
LF/HF	3.28 (1.98 to 4.59)	3.34 (2.22 to 4.45)	3.81 (2.1 to 5.52)	0.54	NA	NA	NA

HRV: heart rate variability; HF: high frequency; LF: low frequency.

Normalized HF: HF/(HF + LF); normalized LF: LF/(HF + LF).

B (baseline): five minutes before massage; D (during massage): the mean of five-minute intervals during massage process; A (after massage): five minutes after massage.

## Abbreviations

ANS: Autonomic nervous system
F&H massage: Foot and hand massage
HF: High frequency
HRV: Heart rate variability
LF: Low frequency
MODS: Multiple organ dysfunction syndrome
PICU: Pediatric intensive care units
PNS: Parasympathetic nervous system
SIRS: Systemic inflammatory response syndrome
SNS: Sympathetic nervous system
VLF: Very low frequency.
